# Ambivalent roles of carboxypeptidase B in the lytic susceptibility of fibrin^☆^

**DOI:** 10.1016/j.thromres.2013.09.017

**Published:** 2014-01

**Authors:** András Kovács, László Szabó, Colin Longstaff, Kiril Tenekedjiev, Raymund Machovich, Krasimir Kolev

**Affiliations:** a Department of Medical Biochemistry, Semmelweis University, Budapest, Hungary; b Institute of Materials and Environmental Chemistry, Research Centre for Natural Sciences, Hungarian Academy of Sciences, Budapest, Hungary; c Biotherapeutics, Haemostasis Section, National Institute for Biological Standards and Control, South Mimms, Potters Bar, UK; d IT Department, N.Y. Vaptsarov Naval Academy, Varna, Bulgaria

**Keywords:** CPB, carboxypeptidase B, CPN, carboxypeptidase N, TAFI, thrombin activatable fibrinolysis inhibitor, tPA, tissue-type plasminogen activator, Carboxypeptidase, Fibrin, Fibrinolysis, Plasmin, tPA

## Abstract

**Background:**

Removal of C-terminal lysine residues that are continuously exposed in lysing fibrin is an established anti-fibrinolytic mechanism dependent on the plasma carboxypeptidase TAFIa, which also removes arginines that are exposed at the time of fibrinogen clotting by thrombin.

**Objective:**

To evaluate the impact of alterations in fibrin structure mediated by constitutive carboxypeptidase activity on the function of fibrin as a template for tissue plasminogen activator-(tPA) induced plasminogen activation and its susceptibility to digestion by plasmin.

**Methods and results:**

We used the stable carboxypeptidase B (CPB), which shows the same substrate specificity as TAFIa. If 1.5 – 6 μM fibrinogen was clotted in the presence of 8 U/mL CPB, a denser fibrin network was formed with thinner fibers (the median fiber diameter decreased from 138 – 144 nm to 89 – 109 nm as established with scanning electron microscopy). If clotting was initiated in the presence of 5 – 10 μM arginine, a similar decrease in fiber diameter (82 -95 nm) was measured. The fine structure of arginine-treated fibrin enhanced plasminogen activation by tPA, but slowed down lysis monitored using fluorescent tPA and confocal laser microscopy. However, if lysis was initiated with plasmin in CPB-treated fibrin, the rate of dissolution increased to a degree corresponding to doubling of the plasmin concentration.

**Conclusion:**

The present data evidence that CPB activity generates fine-mesh fibrin which is more difficult to lyse by tPA, but conversely, CPB and plasmin together can stimulate fibrinolysis, possibly by enhancing plasmin diffusion.

## Introduction

Binding of tissue-type plasminogen activator (tPA) and plasminogen to fibrin is a prerequisite for efficient fibrinolysis (reviewed in [1]), in the course of which the generated plasmin provides a positive feed-back loop through exposure of new carboxyl terminal lysines that promote fibrinolysis primarily through plasminogen and plasmin binding [2]. The amplifying effect of C-terminal lysines on plasminogen activation and the protection of the bound plasmin against its major plasma inhibitor α_2_-plasmin inhibitor is counterbalanced by the action of thrombin activatable fibrinolysis inhibitor (TAFI, or carboxypeptidase U), an exopeptidase that removes basic amino acids (arginine and lysine) from the C-terminal of peptides (reviewed in [3]). TAFI is present in blood plasma in zymogenic form and it is activated by thrombin-thrombomodulin complex to TAFIa, but its activity decays with a half-life of several minutes [4]. A distinct, constitutively active enzyme, carboxypeptidase N (CPN) is also present in plasma [5], [6]. It binds fibrin and can be detected in the structure of plasma clots [7], [8]. Despite the identical primary specificity of TAFIa and CPN the anti-fibrinolytic action of CPN is only a fraction of that of TAFIa even after an activating cleavage by plasmin [9]. The lysis of whole blood clots immersed in tPA is surprisingly insensitive to the action of CPN; no impairment of lysis is observed under conditions when CPN removes lysyl residues from fibrin at a rate corresponding to 50% of that by TAFIa [10]. The background of the differential anti-fibrinolytic potential of CPN and TAFI is still not clarified.

Another determinant of fibrinolytic efficiency is fibrin structure, which as a cofactor for tPA, regulates plasminogen activation, and susceptibility to plasmin digestion (reviewed in [11]). The initial structure and subsequent rearrangements of fibrin during lysis mean that binding events, plasminogen activation and fibrin digestion can be affected in complex ways, sometimes in opposite directions [12]. With this in mind, a question arises, can carboxypeptidase activity expressed during clot formation and dissolution induce structural changes that modulate fibrinolysis? The timeliness of this question also stems from rather contradictory data on the association of TAFI and thrombotic events reported in epidemiological studies. TAFI levels in circulation are regulated by known gene polymorphisms, but the connection with coronary heart disease [13] or stroke [14] is less clear. In the absence of any mechanistic evidence for antithrombotic effects of TAFI the controversy that both high and low thrombotic risk can be accompanied by elevated TAFI levels in plasma [15], [16], [17], [18], [19] is currently explained by the different methods used for determination of TAFIa activity or TAFI antigen and the different sensitivity of the detection assays for TAFI isoforms [20]. However, additional levels of complexity may still remain uncovered as suggested by the fact that some aspects of TAFIa mechanism of action established *in vitro* remain a puzzle. For example, if fibrin containing α_2_-plasmin inhibitor or plasma clots are supplemented with increasing concentrations of TAFIa, a threshold concentration can be reached beyond which TAFIa is profibrinolytic [21]. One hypothesis for this phenomenon is the removal of lysine^452^ of α_2_-plasmin inhibitor by carboxypeptidases [21], but this is difficult to reconcile with the findings that the C-terminal lysine in the inhibitor is not essential for its interaction with plasmin [22]. The completely normal phenotype of TAFI knock-out mice (including hemostasis at basal state or if challenged by a variety of prothrombotic stimuli) [23] also raises the possibility for subtle TAFI effects *in vivo* beyond the known mechanism of action. Thus, the details of TAFI function still require further elaboration despite its well-documented anti-fibrinolytic action *in vitro*. The present study addresses the modulation of fibrinolysis related to fibrin structure which is altered as a consequence of basic carboxypeptidase activity.

## Materials and methods

Plasminogen-depleted human fibrinogen, streptokinase and porcine carboxypeptidase B (CPB) were from Calbiochem (La Jolla, CA). The chromogenic substrate for plasmin, Spectrozyme-PL (H-D-norleucyl-hexahydrotyrosyl-lysine-p-nitroanilide) was from American Diagnostica (Pfungstadt, Germany) and tPA was from Boehringer Ingelheim (Germany). Bovine thrombin was purchased from Serva (Heidelberg, Germany) and further purified by ion-exchange chromatography on sulfopropyl-Sephadex yielding a preparation with specific activity of 2100 IU/mg [24] and 1 IU/mL was considered equivalent to approximately 10.7 nM by active site titration [25]. Alexa Fluor® 546-conjugated fibrinogen was the product of Invitrogen Life Technologies, Budapest, Hungary. Human plasminogen was purified by affinity chromatography on Lysine-Sepharose from citrated human plasma provided by the Hungarian Blood Supply Service [26]. The generation of plasmin from the zymogen and determination of its active concentration were performed as previously described [27]. Blood was collected from healthy volunteers with venipuncture in 10 mM trisodium-citrate (final concentration) and following 10-min centrifugation at 2,000 *g* the top ¾ of the plasma layer was used for the measurements within 4 h.

### Turbidimetric fibrinolytic assays

In 96-well microtiter plates, 6 μM fibrinogen in 10 mM HEPES buffer pH 7.4 containing 150 mM NaCl and arginine or CPB at various concentrations were mixed with 20 nM thrombin in a total volume of 100 μl. In the assays when lysis was initiated by surface tPA, fibrinogen also contained 1 μM plasminogen and following 30 min clotting, tPA was applied to the surface of clots at 15 nM. The concentration of CPB that produced maximum effect in this assay (8 U/mL) was applied in the rest of the experiments in this study. In the assays when lysis was initiated by tPA dispersed in the clot, fibrinogen contained 0.25 μM plasminogen and 0.1 nM tPA was added together with thrombin. In the assays when lysis was initiated by plasmin its concentrations were chosen to yield complete dissolution within 5 h; in the range 2-10 nM for plasmin uniformly dispersed in the clot and 0.5 – 2 μM for plasmin applied to the clot surface as described previously [28], [29]. Clot formation and dissolution was followed by measuring the light absorbance at 340 nm at 37 °C with a Zenyth 200rt microplate spectrophotometer (Anthos Labtec Instruments GmbH, Salzburg, Austria). For adequate comparison of lytic rates from measurements, in which different maximum turbidity values were reached despite the identical quantities of fibrin, the absorbance values were evaluated in normalized form [30]. The time needed to reduce the turbidity of the clot to a given fraction of the maximal value (*t_0.5_* to reach 0.5*A_max_*, *t_0.1_* reach 0.1*A_max_*) was used as a quantitative parameter of fibrinolytic activity. In certain cases plasma containing various concentrations of added arginine and CPB was clotted with 15 nM thrombin and 12.5 mM CaCl_2_. If plasma clot dissolution was mediated by tPA dispersed in the clot, tPA was added at 0.8 nM prior clotting, whereas the concentration of tPA applied to the surface of plasma clots was 30 nM.

### Scanning electron microscopic (SEM) studies

Fibrin clots of 50 μl volume were prepared in duplicate: fibrinogen (at concentration in the range 1.5 – 6 μM) in 10 mM HEPES buffer pH 7.4 containing 150 mM NaCl and the additives (arginine or CPB) was clotted with 20 nM thrombin at 37 °C for 30 min. Thereafter clots were placed into 10 mL 100 mM Na-cacodylate pH 7.2 buffer for 24 h at 4 °C. Following repeated washes with the same buffer, samples were fixed in 1%(v/v) glutaraldehyde for 16 h. The fixed samples were dehydrated in a series of ethanol dilutions (20 – 96%(v/v)), 1:1 mixture of 96%(v/v) ethanol/acetone and pure acetone followed by critical point drying with CO_2_ in E3000 Critical Point Drying Apparatus (Quorum Technologies, Newhaven, UK). The specimens were mounted on adhesive carbon discs, sputter coated with gold in SC7620 Sputter Coater (Quorum Technologies, Newhaven, UK) and images were taken with scanning electron microscope EVO40 (Carl Zeiss GmbH, Oberkochen, Germany). The SEM images were analyzed to determine the diameter of the fibrin fibers using self-designed program functions running under the Image Processing Toolbox v. 8.2 of Matlab 8.1.0.604 (R2013a) (The Mathworks, Natick, MA) as previously described [12], [31].

### Plasminogen activation assay

In 96-well microtiter plates, 6 μM fibrinogen in 10 mM HEPES buffer pH 7.4 containing 150 mM NaCl, 0.5 μM plasminogen and additives (arginine or CPB) was clotted with 25 nM thrombin in a volume of 80 μl. After 30 min at 37 °C 60 μl of 15 nM tPA and 0.6 mM Spectrozyme-PL in 10 mM HEPES, 150 mM NaCl pH 7.4 were placed on the surface of the clot. The forming plasmin generated p-nitroaniline, the absorbance of which was continuously recorded at 405 nm (A_405_) with Zenith 200rt spectrophotometer. The measured values were plotted versus time squared (*t^2^*) yielding a linear relationship according to the equation *ΔA_405_ = 0.5εk_1_k_cat_[tPA] t^2^* [32], where *ε* = 12.6 mM^- 1^ cm^- 1^ is the extinction coefficient of p-nitroaniline [33], *k_1_* = 350 min^- 1^ is the turn-over number of plasmin on Spectrozyme-PL [33], *k_cat_* and *[tPA]* are the catalytic constant for plasminogen activation and the concentration of tPA in the reactive layer on the surface of fibrin, respectively [34]. The term *V_app_ = k_cat_[tPA]* is equivalent to the apparent maximal rate of plasminogen activation in the reactive layer of fibrin and was determined from linear regression according to the abovementioned equation (Curve fitting toolbox v. 3.3.1 of Matlab 2013a).

### Confocal microscopic imaging

Fibrin clots were prepared from 6 μM fibrinogen, 2% of which was Alexa Fluor® 546-labelled in 10 mM HEPES buffer pH 7.4 containing 150 mM NaCl, 1.5 μM plasminogen and the tested additives with 15 nM thrombin for 30 min at room temperature in sterile, uncoated IBIDI VI 0.4 μ-slides (Ibidi GmbH, Martinsried, Germany). Thereafter 50 nM tPA-YFP (tPA with Yellow Fluorescent Protein fused to its C-terminal expressed using pFastBac-tPA as previously described) [12] was added to the edge of the clot and the fluorescence (excitation wavelength 488 nm, emission wavelength 525 nm for tPA-YFP detection and excitation wavelength 543 nm, emission wavelength 575 nm for Alexa546-fibrinogen detection) was monitored with Confocal Laser Scanning System LSM710 (Carl Zeiss GmbH, Jena, Germany) taking sequential images of the fluid-fibrin interface at a distance of approximately 50 μm from the chamber surface with identical exposures and laser intensities using a Plan-Neofluar 20x/0.5 objective. It should be noted that the 10 – 20 μm fluorescent aggregates present in the commercial Alexa Fluor® 546-labelled fibrinogen were not centrifuged before clotting as done in our earlier work [35], but these were preserved and used as position markers in the fibrin.

### Statistical analysis

The distribution of the data on fiber diameter was analyzed according to an algorithm used previously [12], [31]: theoretical distributions were fitted to the empirical data sets and compared using Kuiper test and Monte Carlo simulation procedures. The statistical evaluation of other experimental measurements in this report was performed with Kolmogorov-Smirnov test (Statistics Toolbox 8.2 of Matlab R2013a).

## Results

### Structural modifications of fibrin related to CPB activity or presence of arginine

**Fig. 1:**
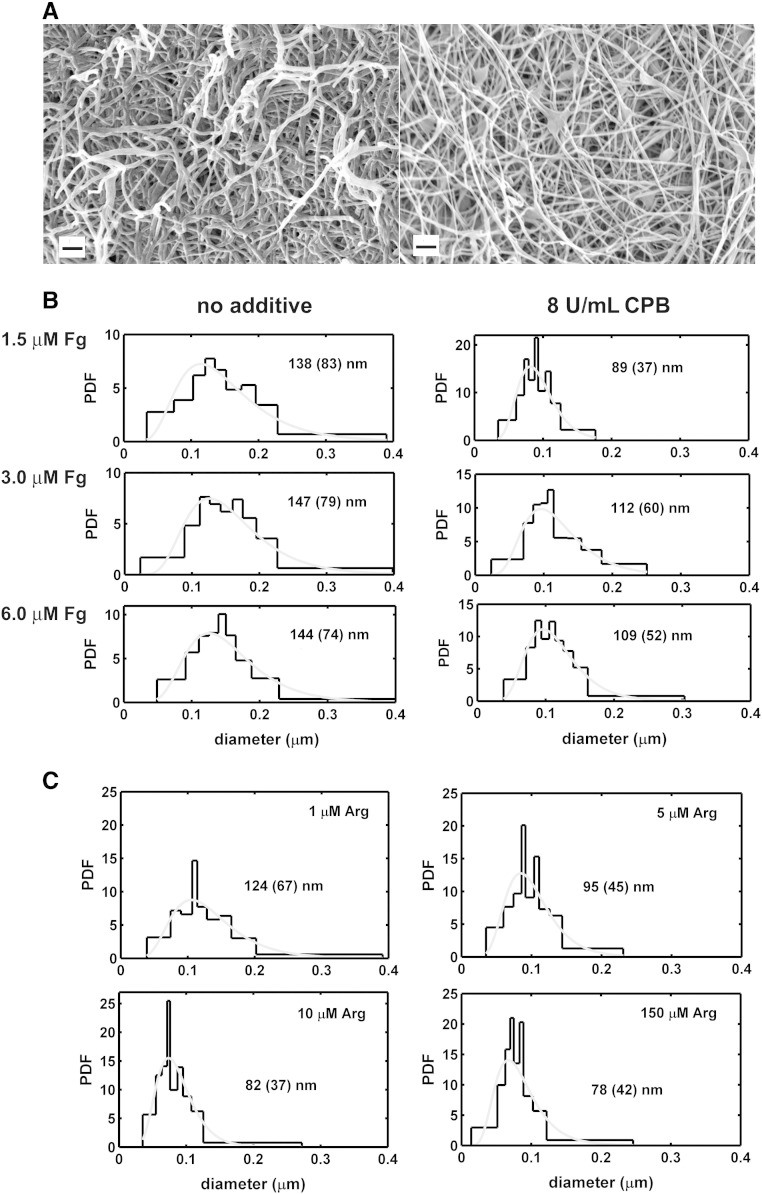
**Modification of fibrin structure by arginine and carboxypeptidase B.** Fibrin clots were prepared in duplicate from fibrinogen at various concentrations and following fixation and drying images were taken with a scanning electron microscope (SEM). A: Representative images of native fibrin at 6 μM (left panel) and fibrin at the same concentration treated with 8 U/mL CPB. Scale bar = 1 μm. B: Effect of CPB on the ultrastructure of fibrin. Morphometric analysis of fibrin was performed on SEM images illustrated in panel A. The diameter of 300 fibres was measured and their empiric (black histograms) as well as best-fitted theoretical (gray curves) probability density function (PDF) was determined. Median values and interquartile range (in brackets) are shown for the theoretical distributions of the diameter values. The concentrations of fibrinogen (Fg) indicated on the left side refer to both non-treated and CPB-treated fibrin. C: Effect of arginine on the fibrin structure. The same morphometric analysis was performed on fibrin prepared from 6 μM fibrinogen containing the concentrations of arginine indicated.

We have previously shown [12], when CPB was used as a stable analogue of TAFIa to evaluate the impact of removal of carboxyl terminal lysines on the kinetics of fibrinolysis, that the presence of CPB during fibrinogen clotting modifies the turbidity of the clots before initiation of lysis, which suggested changes in the structure of fibrin. SEM imaging provided direct evidence for the alteration of the fibrin structure related to the action of CPB (Fig. 1A). Morphometric analysis of the SEM images (Fig. 1B) revealed that the fiber diameter decreased by 21 – 25% in CPB-treated clots prepared from fibrinogen at physiologically relevant concentration (1.5 – 6 μM). Because the native fibrinogen molecule does not contain any substrate (C-terminal lysine or arginine) for CPB, the single target of CPB action in this purified system could be the arginine residues in the fibrinopeptides newly cleaved by thrombin in the process of clotting. If all new C-terminal arginines were released from the 4 fibrinopeptides derived from each fibrinogen molecule, an increment of 25 – 50 μM would be expected in the local concentration of arginine in clots prepared from 2 – 4 mg/mL fibrinogen (a prediction supported by direct measurements of arginine release by TAFIa in fibrin: 10 μM in 5 min after clotting [36]). As evidenced by the morphometric data (Fig. 1C), addition of arginine at 5 – 20% of this maximal concentration caused changes in the structure of fibrin comparable to those induced by the action of CPB (Fig. 1B). Considering the normal plasma level of arginine of about 100 μM [37] and these subtle structural alterations in fibrin, inclusion of micromolar arginine appears to be essential for appropriate modelling of the physiological conditions when fibrin structure-dependent processes are evaluated with *in vitro* assays.

### Kinetics of plasminogen activation and fibrin dissolution

**Fig. 2:**
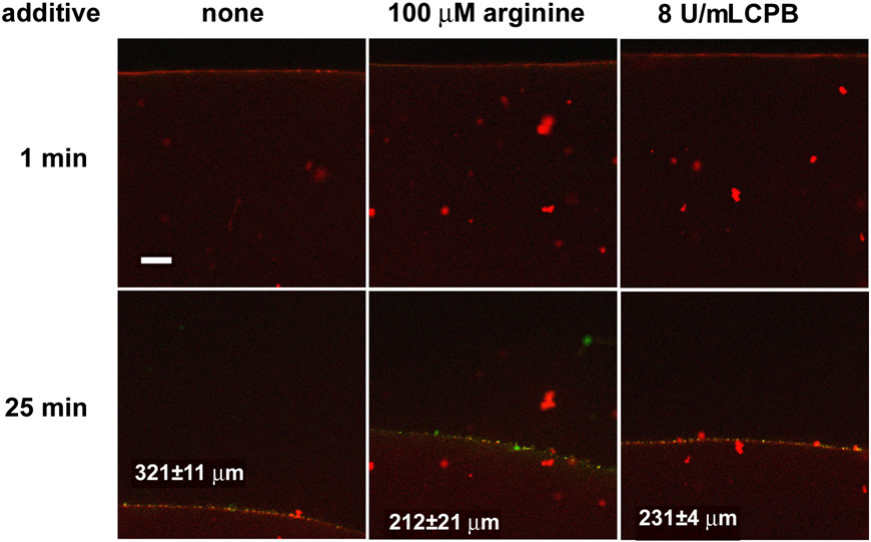
**Effects of arginine and carboxypeptidase B on the penetration of tPA-YFP into fibrin in the course of lysis.** Clots were prepared from fibrinogen containing Alexa^546^-label, plasminogen and the indicated additives. tPA-YFP was added to the surface of fibrin (at the top of the images) and the fluid/fibrin interface was monitored by confocal laser scanning microscopy using double fluorescent tracing (tPA-related fluorescence stains in green, whereas the fibrin is shown in red in these images). The time after the application of tPA-YFP is indicated, and the scale bar = 50 μm. The numbers in the bottom panels indicate the distance for penetration of tPA-YFP in the clot at 25 min (mean and standard deviation from 3 samples, the values for arginine and CPB differ from pure fibrin at *p* < 0.05 level according to Kolmogorov-Smirnov test).

**Fig. 3:**
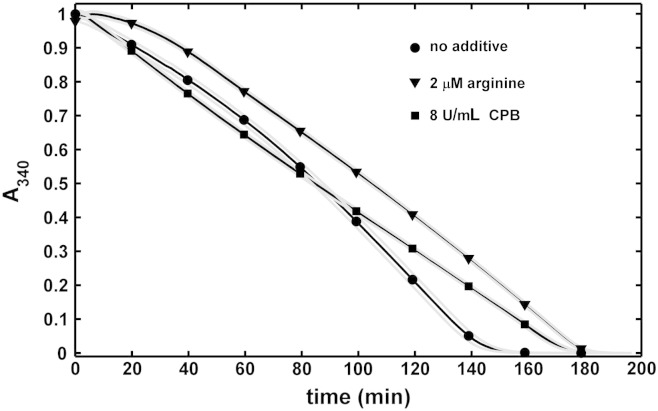
**Arginine and carboxypeptidase B in fibrin clot lysis assay.** Fibrin clots containing plasminogen and the indicated additives were prepared, tPA was added to the surface and the absorbance was continuously measured at 340 nm (turbidity, A_340_ is presented in relative units, normalized for maximal value of absorbance of each individual curve). Mean values of 8 measurements from 3 independent experiments (continuous lines with symbols at every 10th measured point for identification of the curves) and SEM values above and below mean (gray lines) are shown.

**Fig. 4:**
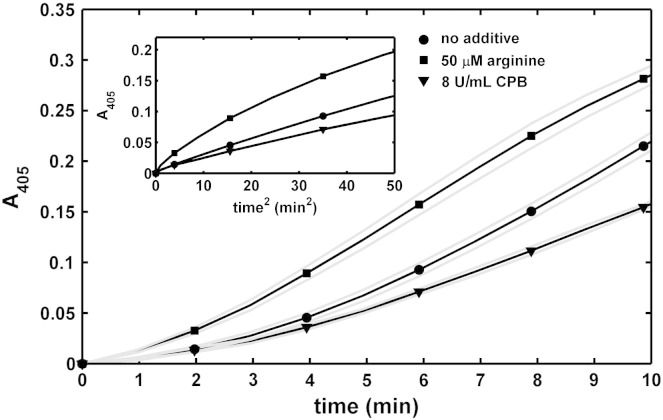
**Plasminogen activation on the surface of fibrin.** Fibrin clots containing plasminogen and the indicated additives were prepared and following addition of tPA and the plasmin substrate Spectrozyme-PL the absorbance was continuously measured at 405 nm (A_405_). Mean values of 8 measurements from 3 independent experiments (continuous lines with symbols at every 5th measured point for identification of the curves) and SEM values above and below mean (gray lines) are shown. Inset: Secondary plots of the raw data the slopes of which represent the apparent maximal activation rates of plasminogen as defined in Materials and methods (1.1 nM/min in the absence of additives, 0.8 nM/min in CPB-treated fibrin, 1.8 nM/min in arginine-modified fibrin).

**Fig. 5:**
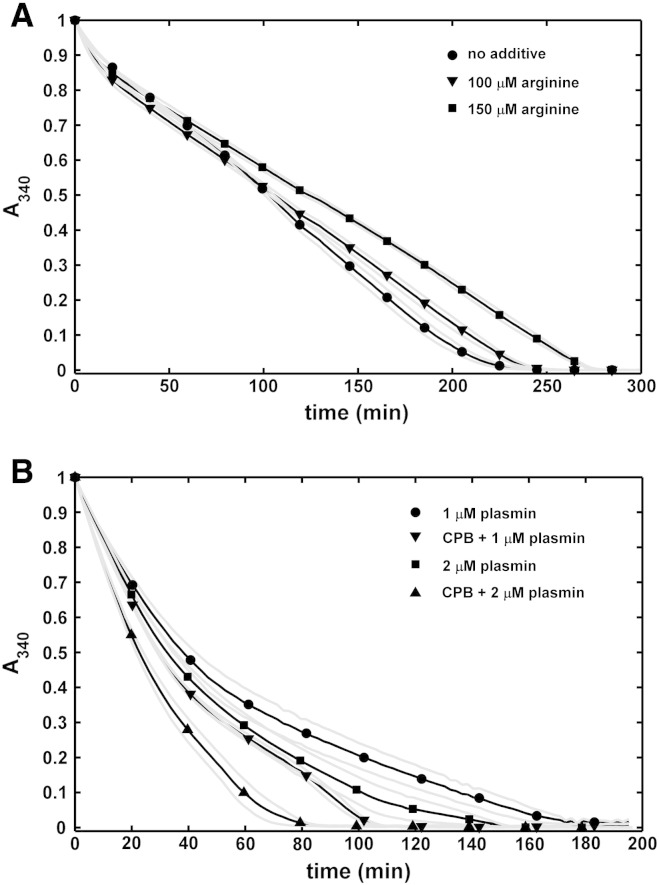
**Dissolution of fibrin by plasmin applied to the surface of the clots.** Fibrinogen containing the indicated additives was clotted with thrombin and thereafter plasmin was applied to its surface and the absorbance was continuously measured at 340 nm (turbidity, A_340_ is presented in relative units, normalized for maximal value of absorbance of each individual curve). Mean values of 8 measurements from 3 independent experiments (continuous lines with symbols at every 10th measured point for identification of the curves) and SEM values above and below mean (gray lines) are shown. A: Effects of arginine on lysis by 0.5 μM plasmin. B: Effect of 8 U/mL CPB on the lysis of fibrin by plasmin.

On a microscopic scale (Fig. 2), tPA-induced lysis of fibrin modified by CPB was significantly slowed down with a granular pattern of accumulation of tPA on the surface of the clot similar to that of native fibrin. On a macroscopic scale of lytic kinetics, CPB had a maximal inhibitory effect at 8 U/mL in the assay when tPA was added to the surface of pre-formed clots containing plasminogen, as shown in Fig. 3. A similar level of inhibition of lysis was also seen in clots formed in the presence of arginine at concentration as low as 2 μM. Although with either CPB or arginine the acceleration seen at later stages of lysis in pure fibrin was missing and despite the identical time to complete dissolution, the lysis kinetics were somewhat different with CPB or arginine, suggesting a different mechanism of action. To address this difference two assays were used; one that monitored solely the generation of plasmin (Fig. 4) and a second one that bypassed the stage of plasminogen activation (Fig. 5).

The effects of CPB and arginine on plasminogen activation by tPA on the surface of fibrin was studied in isolation using the substrate Spectrozyme-PL to follow plasmin generation. As expected, plasmin generation was inhibited in the presence of CPB, presumably resulting from removal of newly exposed C-terminal lysine binding sites in fibrin (Fig. 4). In contrast however, fibrin formed in the presence of arginine proved to be a better template for plasminogen activation than the native fibrin structure (Fig. 4), despite the overall inhibiting effect of arginine on tPA-induced fibrin degradation seen in Fig. 3. It is noteworthy that arginine at identical concentrations does not affect plasminogen activation by tPA in fibrin-free systems (data not shown).

When plasmin was added to the surface of fibrin, slower lysis was observed similarly to the tPA-induced lysis, if clots were formed in the presence of arginine (Fig. 5A). Quite unexpectedly, CPB rendered fibrin more susceptible to lysis by plasmin added to the surface of the clot (Fig. 5B). The complete lysis of CPB-treated fibrin was faster (Table 1) and non-treated fibrin was dissolved by 2 μM plasmin at approximately the same rate as CPB-modified fibrin by 1 μM plasmin (no significant difference between the values for these two states in the 4th and 5th column of Table 1). However, if plasmin was homogeneously dispersed within the clot, neither inhibitory effect of arginine, nor stimulatory effect of CPB could be observed (data not shown). The discordant effects of the modulators in the two assay formats indicate that at least in part their effects arise from the variations in the penetration of plasmin in fibrin of different structure.

**Table 1:** Impact of carboxypeptidase B on the kinetics of fibrinolysis by plasmin. Fibrin clots were prepared and lysis initiated with plasmin at various concentrations as illustrated in Fig. 5B. The time needed for a decrease in the maximal absorbance to the fraction values shown in the indices of *t* is presented in min as mean and SD of 8 measurements from 3 independent experiments (asterisk indicates differences between values for CPB-treated and non-treated samples at *p* < 0.05 level according to Kolmogorov-Smirnov test).

	0.5 μM plasmin		1.0 μM plasmin		2.0 μM plasmin	
CPB	0	8 U/mL	0	8 U/mL	0	8 U/mL
*t_0.9_*	7.9 ± 0.7	10.4 ± 0.6*	5.8 ± 0.7	3.8 ± 0.3*	5.4 ± 1.4	3.9 ± 0.2
*t_0.5_*	75.4 ± 6.7	81.8 ± 2.3	46.5 ± 0.8	37.0 ± 3.5*	33.1 ± 5.6	23.0 ± 2.6*
*t_0.3_*	126.0 ± 9.3	126.3 ± 1.2	77.6 ± 0.9	68.8 ± 4.0*	54.4 ± 11.1	35.6 ± 6.4*
*t_0.1_*	187.0 ± 9.0	165.0 ± 2.6*	126.5 ± 4.1	110.5 ± 3.4*	90.6 ± 19.2	54.5 ± 8.8*

### Fibrinolysis in plasma environment

**Fig. 6:**
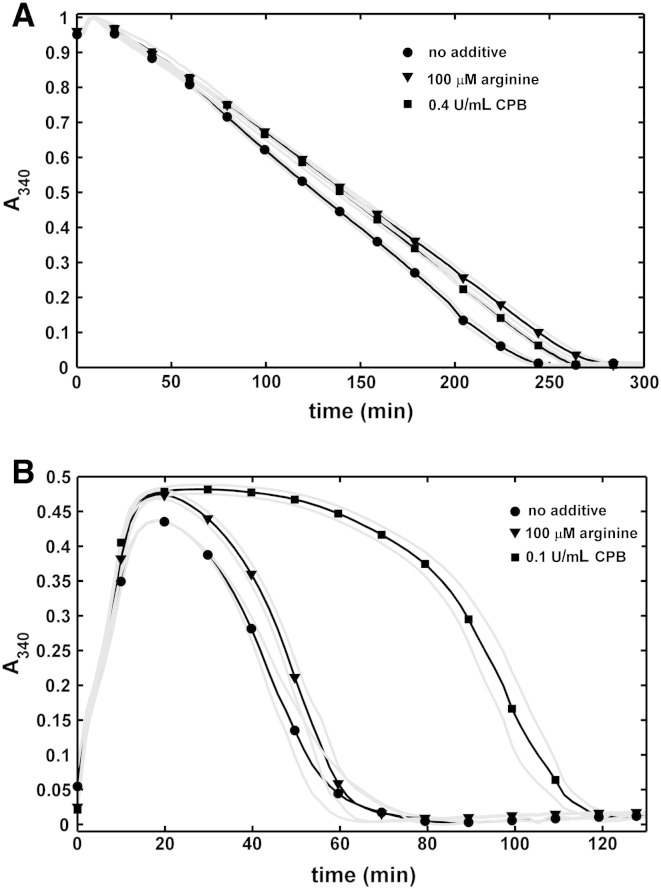
**Effects of arginine and carboxypeptidase B on fibrinolysis in plasma environment.** Citrated plasma containing the indicated additives was clotted with thrombin and recalcification and fibrinolysis was initiated either by 30 nM tPA added to the surface of pre-formed clots (A) or by 0.8 nM tPA mixed with thrombin prior clotting (B). Thereafter the absorbance was continuously measured at 340 nm (A_340_). Mean values of 4 measurements from 3 independent experiments (continuous lines with symbols at every 10th measured point for identification of the curves) and SEM values above and below mean (gray lines) are shown.

In line with the effects observed with purified fibrinolytic components (Fig. 3), arginine and CPB retarded the dissolution of plasma clots by tPA (Fig. 6). However, CPB inhibited plasma clot lysis at lower concentrations than used in the purified systems above. If tPA was uniformly dispersed in the plasma prior clotting, 0.1 U/mL CPB prolonged the half lysis time more than two-fold, whereas the effect of arginine at physiologically relevant concentration (100 μM) was minimal (Fig. 6B). If tPA was applied to the surface of pre-formed clots, the effect of this arginine was equivalent to 0.4 U/mL CPB (Fig. 6A).

## Discussion

Since the discovery of TAFI [38] its anti-fibrinolytic action has been largely attributed to the removal of plasmin-generated C-terminal lysines in the fibrin matrix, but it has been difficult to show convincingly that TAFI levels affect myocardial infarction or stroke outcomes at a population level or to explain the different anti-fibrinolytic impact of TAFI and plasma CPN. In the present study we used CPB, a homologous, but stable carboxypeptidase with specificity for basic amino acids and addressed alternative or additional factors that could modulate its anti-fibrinolytic function. The application of CPB overcomes the short half-life of TAFIa of approximately 10 min [4], has the technical advantage that it does not inactivate plasmin in contrast to TAFIa [36] and models also the constitutive *in vivo* activity of CPN at the stage of fibrin clot formation. Our work identified two consequences of CPB activity in fibrin that antagonize the CPB-dependent blockade of the positive feed-back loop in fibrinolysis based on C-terminal lysine exposure: 1) enhancement of plasmin activity when the fluid-borne enzyme attacks the surface of pre-formed clots, probably due to improved diffusion; and 2) fibrin structure-related acceleration of plasminogen activation.

### Degradation of fibrin by plasmin at the surface of the clot

A rather unexpected observation arose from the study of surface application of plasmin to fibrin containing CPB (Fig. 5B, Table 1), where increased potency of plasmin was noted. Under the conditions shown, CPB treatment was equivalent to doubling the concentration of plasmin. This was in contrast to the lack of CPB effect on fibrin digestion when plasmin was dispersed in the clot (data not shown). Enhanced susceptibility to plasmin could be explained by removal of newly exposed C-terminal lysines that serve as binding sites for plasmin and retard its penetration into the lysing clot. The elimination of these retarding interactions shifts the dynamic equilibrium of free and bound plasmin in favour of the former which is actually engaged in the degradation of the fibrin matrix. This interpretation of the CPB effect on plasmin-mediated fibrinolysis is in agreement with earlier findings that TAFIa increases the susceptibility of fibrin-bound plasmin to inactivation by α_2_-plasmin inhibitor [39] due to elimination of binding sites essential for the reduction of the inhibition rate constant by orders of magnitude compared to fibrin-free solution [27]. This enhancement of plasmin action provides an explanation for the observed reversal of the anti-fibrinolytic effect of TAFI in situations when abundant plasmin is formed at the surface of the clot and thus the major control point of the overall fibrinolysis is shifted from the stage of plasminogen activation to the action of plasmin. Such a loss of TAFI-dependent inhibition of fibrinolysis has been reported for high concentrations of tPA [40] and other activators [41], as well as when plasma is supplemented with plasminogen [42]. The key finding from the present study that CPB activity favours the action of plasmin is in line with the predictions of recent theoretical models of fibrinolysis [43] and provides a clue for understanding the difference in the anti-fibrinolytic potential of a transiently active (TAFIa) and constitutive (CPN) carboxypeptidase [9], [10].

### tPA-mediated fibrinolysis

Although as expected, CPB slows down the tPA-dependent lysis of both pure fibrin (Fig. 2, Fig. 3) and plasma clots (Fig. 6), the route of action of the activator (at the surface or within the clot) profoundly affects its sensitivity to the action of CPB. The difference in the fibrinolytic outcome for the two accession routes could be explained by the diffusion-dependent effects of CPB on plasmin as discussed above (the favorable CPB effects on plasmin at the clot surface counteract the inhibition of plasminogen activation, whereas in the absence of such a counter-balancing factor lower CPB activity is sufficient to inhibit the tPA-mediated lysis in a homogenous assay format). In addition, we observed that plasminogen activation on fibrin by fluid-borne tPA is less affected than expected from earlier studies [36]. In different experimental setups, where unidirectional diffusion or matrix penetration is not at issue (e.g. with enzymes uniformly dispersed within fibrin clots), the cleavage of C-terminal lysines reduces the rate constant of plasminogen activation by a factor of 2.5 [36]. In contrast, only a 27% decrease in the plasminogen activation rate on the surface of fibrin can be achieved by CPB treatment (Fig. 4). This discrepancy may be explained by a compensatory increase in plasminogen activation rate due to the formation of fine mesh fibrin related to CPB activity.

Interestingly, the same structural alterations were observed in the presence of arginine at micromolar concentrations (Fig. 1C). Because in the concentration range up to 250 μM, arginine does not affect plasmin activity on a small peptide substrate (Spectrozyme PL) and plasminogen activation by tPA in fibrin-free systems (data not shown), measurements with arginine-modified fibrin proved to be a helpful tool to discriminate between cleavage of C-terminal lysines and modification of fibrin structure as a background of the CPB effects discussed above. The minimal inhibitory effect of arginine-modified fibrin on plasmin action at the surface (Fig. 5A) precludes a role for fine-mesh structure in the pro-fibrinolytic effect of CPB (Fig. 5B) and thus supports the interpretation of the results based on changes in the penetration pattern of plasmin in fibrin devoid of C-terminal lysines. The definite acceleration of plasminogen activation on arginine-modified fibrin (Fig. 4) supports the conclusion given above that the observed rate of plasmin generation on CPB-modified fibrin is the outcome of two opposing effects; a positive one based on fine-mesh structure and a negative one based on elimination of C-terminal lysine binding sites that would otherwise promote plasminogen activation.

Out of these findings a hypothesis is emerging for a causative relationship between carboxypeptidase activity, arginine release and structure/function alterations of fibrin. Variations of arginine concentration within the range used in the present study could arise *in vivo* from TAFIa action on fibrinopeptides A and B released by thrombin as reported earlier by direct arginine measurements in fibrin clots prepared from physiologically relevant fibrinogen concentrations [36]. Alternative sources of carboxypeptidase activity at the stage of fibrin formation (before TAFIa peaks) could be the constitutive plasma CPN and under pathological conditions even pancreatic CPB [44]. A fraction of the arginine concentration that can be maximally released from this source was sufficient to modify the structure of fibrin in a similar way to CPB (Fig. 1). It is noteworthy that in the course of tPA-mediated lysis of pure fibrin, TAFIa releases both arginine and lysine at increasing concentrations in the micromolar range and a ratio that is consistently about five-fold in favor of arginine over a period of several hours [36]. This fact has been largely ignored in the *in vitro* fibrinolytic studies using clots prepared from purified fibrinogen or diluted plasma. Because the results of the present study indicate that the effects of arginine on fibrin structure and fibrinolysis are saturable at concentrations approaching the physiological plasma level of 100 μM [37], and in vitro the generation of arginine up to 50 μM may account for variability related to the application of various concentrations of TAFIa (or CPB).

### In vivo considerations

The normal plasma concentration of arginine of about 100 μM provides a baseline level of arginine, at which blood clots are formed *in vivo*. However, in sepsis the systemic arginine concentration falls to values below 50 μM [45], which means that locally, around inflammatory cells expressing inducible NO synthase, arginine could be severely depleted. Furthermore, red blood cells contain high concentrations of arginase I [46], which could deplete the arginine in erythrocyte-rich thrombi. Thus, depending on the cell content of thrombi the arginine concentrations can vary over a range capable of producing the effects evaluated in the present study.

Our study demonstrates that carboxypeptidase-related modulation of fibrinolysis is more complex than generally believed. To date, down regulation of fibrinolysis *in vivo* has been ascribed to bypassing of the positive feed-back loop in plasminogen activation or accelerated inactivation of plasmin by α_2_-plasmin inhibitor in the lysing fibrin. The current study suggests inhibition of fibrinolysis may be counteracted to some degree by carboxypeptidases (TAFIa, CPN) effecting changes in fibrin ultrastructure to improve plasminogen activation rates and by enhancing plasmin diffusion in fibrin clots. Consideration of these subtle effects and their balance appears essential in view of the recent interest in TAFIa as a target of pharmacological agents for potential administration as standalone or adjuvant thrombolytics (reviewed in [47], [48]) and the previously suggested regulatory role for the plasma CPN under certain conditions [9], [49].

## Conflict of interest statement

None to declare.
